# Patient-centric synthetic data generation, no reason to risk re-identification in biomedical data analysis

**DOI:** 10.1038/s41746-023-00771-5

**Published:** 2023-03-10

**Authors:** Morgan Guillaudeux, Olivia Rousseau, Julien Petot, Zineb Bennis, Charles-Axel Dein, Thomas Goronflot, Nicolas Vince, Sophie Limou, Matilde Karakachoff, Matthieu Wargny, Pierre-Antoine Gourraud

**Affiliations:** 1 Octopize, Mimethik Data, Nantes, France; 2 Nantes Université, INSERM, CHU de Nantes, Ecole Centrale de Nantes,Centre de Recherche Translationnelle en Transplantation et Immunologie, CR2TI, Nantes, France; 3 Nantes Université, CHU de Nantes, INSERM, CIC 1413, Pôle Hospitalo-Universitaire 11: Santé Publique, Clinique des données, Nantes, France

**Keywords:** Databases, Medical research, Data publication and archiving, Databases

## Abstract

While nearly all computational methods operate on pseudonymized personal data, re-identification remains a risk. With personal health data, this re-identification risk may be considered a double-crossing of patients’ trust. Herein, we present a new method to generate synthetic data of individual granularity while holding on to patients’ privacy. Developed for sensitive biomedical data, the method is patient-centric as it uses a local model to generate random new synthetic data, called an “avatar data”, for each initial sensitive individual. This method, compared with 2 other synthetic data generation techniques (Synthpop, CT-GAN), is applied to real health data with a clinical trial and a cancer observational study to evaluate the protection it provides while retaining the original statistical information. Compared to Synthpop and CT-GAN, the Avatar method shows a similar level of signal maintenance while allowing to compute additional privacy metrics. In the light of distance-based privacy metrics, each individual produces an avatar simulation that is on average indistinguishable from 12 other generated avatar simulations for the clinical trial and 24 for the observational study. Data transformation using the Avatar method both preserves, the evaluation of the treatment’s effectiveness with similar hazard ratios for the clinical trial (original HR = 0.49 [95% CI, 0.39–0.63] vs. avatar HR = 0.40 [95% CI, 0.31–0.52]) and the classification properties for the observational study (original AUC = 99.46 (*s.e*. 0.25) vs. avatar AUC = 99.84 (*s.e*. 0.12)). Once validated by privacy metrics, anonymous synthetic data enable the creation of value from sensitive pseudonymized data analyses by tackling the risk of a privacy breach.

## Introduction

During the past decade, data value and accessibility have increased tremendously^1^. Many private and public institutions generate, analyze and store data on behalf of their stakeholders, users, customers, or patients. Accumulated data are often considered a byproduct of data activity. However, value is also created by re-analyzing, sharing, and eventually licensing out data. Until recently, threats to personal privacy have been considered unavoidable, and the re-identification risk was either unstudied or underestimated^2^. Rocher et al. showed that 99.98% of the people could be re-identified in any pseudonymized dataset using 15 demographic attributes. Other studies involving various data types such as mobility^3–6^, credit card^7^, and browsing data^8^ have shown that de-identification is insufficient to protect personal data^9–11^. Value is too often extracted from data at the expense of privacy. In the health domain, the emergence of biomedical data warehouses and electronic health records has increased attention to data sensitivity. For example, in 2017, Culnane et al.^12^ re-identified a patient from an Australian de-identified open health dataset^13^. The risk of personal data being stolen is high^14^, frequently underestimated and could lead to ransomware in hospitals worldwide^15^. Although data sharing is fundamental for research, re-identification of patients’ health issues^11^ and individually discriminating information is a threat and a limiting factor.

Since 2018, the implementation of the General Data Protection Regulation (GDPR) in Europe has significantly changed the regulatory framework for the circulation and use of personal data, promoting among other things, a more systematic use of anonymization techniques. However, patients, citizens, and scientists alike often mistake pseudonymized data for anonymized data. With pseudonymized data, all directly identifying information (e.g., name, phone number, social security number) has been removed to prevent the risk of direct identification of the patient. However, the risk of re-identification remains and is often unquantified. Pseudonymization is not a type of anonymization^16^. According to Recital 26 of the GDPR^17^, anonymous data are defined as “information which does not relate to an identified or identifiable natural person or to personal data rendered anonymous in such a manner that the data subject is not or no longer identifiable”.

The European Data Protection Board (EDPB) proposes three principles to evaluate the robustness of an anonymization process^16^: (1) Singling out, which corresponds to the possibility of isolating some or all records that identify an individual in a dataset; (2) Linkability, which is the ability to link at least two records concerning the same data subject or group of data subjects (either in the same or different databases), and (3) Inference, which is the possibility to deduce, with a significant probability, the value of an attribute from the values of a set of other attributes. In other words, once anonymized, it is no longer possible to (1) single out a patient within a dataset, (2) match records between different data sources, and (3) deduce the real patient outcome.

To meet these legal and privacy issues, anonymization techniques are worthwhile solutions for data privacy. Scientific research has yielded a range of anonymization techniques (noise addition^18^; substitution; aggregation or K-anonymity^19^; L-diversity^20^; differential privacy^21^; hashing/tokenization^22^). Among them, differential privacy is considered one of the most prominent properties by providing a mathematical proof of the level of privacy with the concept of ε-differential privacy. Yet, its application requires access to the original database and is designed to produce statistics. By mathematically simulating the whole individual observation, synthetic datasets protect individual privacy while attempting to retain the statistical relevance of the dataset. Synthetic data are defined as any production data not obtained from real measurements^23^. In practice, these data are drawn at random using data models whose objective is to mimic a real dataset or an individual observation. Synthetic datasets offer the following advantages^24^: (1) structural similarity (i.e., the same granularity): the synthetic dataset contains the same number of observations, the same number of variables, and the same variable types; (2) information relevance: the analyst will obtain results from the synthetic dataset that are comparable to the original data, and (3) subjective assessment: neither experts nor trained algorithms can distinguish synthetic data from original data. Recent techniques based on computational power enabling machine learning^25–29^ and more accurate efforts of statistical modeling^30–32^ have significantly improved the possibility of creating synthetic data. The simulation of synthetic data is often based on mathematical modeling and fairly well mimics the statistical properties^33^ of the original data; however, the privacy risk is rarely documented^25,26,34^. The simulated nature of synthetic data drawn at random from a model makes the individual privacy risk hard to quantify^35^.

Herein, we present a new algorithm for generating synthetic data called the “Avatar” method. This methodology uses a built-in patient-centered approach. As it uses each sensitive observation to create a local simulation leading to the creation of a single avatar simulation, the synthetic data can be evaluated in light of the three criteria of the EDPB. We compare the Avatar method with two reference techniques using, respectively, classification and regression trees and Generative Adversarial Networks approaches. The methods are applied to two biomedical datasets to illustrate that synthetic data preserve the structure and statistical relevance of the original dataset. The Avatar method maintains a similar level of utility compared to other synthetic data generation methods. The analysis performed on the clinical trial data show similar treatment’s effectiveness (Original Hazard Ratio (HR): 0.49, avatar HR: 0.40, Synthpop HR: 0.59, CT-GAN HR: 0.25). The classification properties of the observational study also remain (Original AUC: 99.46, avatar AUC: 99.84, Synthpop AUC: 99.24, CT-GAN AUC: 99.95). Generic privacy metrics show that the Avatar method generates data on average closer to the original than Synthpop and CT-GAN. None of the methods generate close and isolated original and synthetic pairs. We show that the patient-centric nature of the Avatar method facilitates the computation of privacy metrics that satisfy EDPB criteria while allowing a level of signal maintenance equivalent to the most efficient state-of-the-art methods. Its explainable approach allows data sharing without compromising privacy.

## Results

### Avatar method and comparative preservation of the statistical relevance

**Fig. 1:**
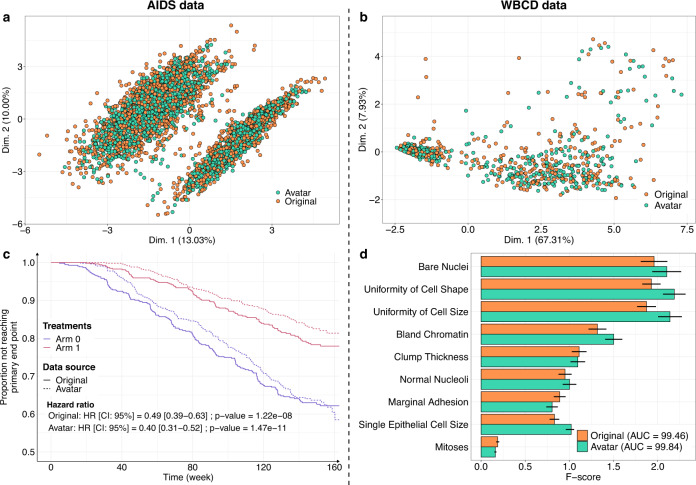
Comparative results of analyses based on original and avatar data. **a**, **b** FAMD projections of the (**a**) AIDS (*k* = 20) and **b** WBCD (*k* = 20) avatar data in the original data space (original data in orange dots, avatar data in green dots). Avatar and original data are overlaid and share the same space built from the original observations. **c** Distributions of times to events were estimated using Kaplan Meier estimate of the time-to event- function and compared with the log-rank test and Cox proportional-hazards model, with a comparison between the original (plain lines) and AIDS avatar data (dotted lines) for arms 0 (purple lines) and 1 (red lines). The statistical *p*-values are computed using Wald test. The original and avatar WBCD datasets were separated into 70 training trials and 30 tests (100 times). **d** Comparison of original (orange bars) and avatar (green bars) *F*-scores for each variable. Error bars represent the 95% confidence interval. SVM machine-learning models were performed using five features selected by *F*-score. The AUC is presented for the original and avatar datasets. Supplementary Tables 1 and 2 show additional statistics. FAMD factor analysis for mixed data, AUC area under the ROC curve, SVM support vector machine, CI confidence interval, HR hazard ratio.

The Avatar method retained the statistical value of the datasets. Figure 1 shows the overlay of the original data (orange) and the avatar data (green). For the AIDS (Fig. 1a) and WBCD (Fig. 1b) datasets, the factor analysis of mixed data (FAMD) projection of the first two components showed that the original data and avatar data fully overlapped, including the outliers. This result indicates that the structure of the information contained in the data has been maintained. Figure 1c compares the survival curves calculated with the avatar dataset and the original AIDS dataset. In both treatment arms, the survival curves of the avatar data (dotted line) and originals (continuous line) overlapped. Regarding the survival curves, the analysis of the avatar data is leading to the same interpretations as the one obtained with sensitive data. Distributions of times to events were estimated with the Kaplan and Meier method and compared with the log-rank test and Cox proportional-hazards model. The statistical *p*-values are computed using Wald test. The main trial results remained unchanged: arm 1 was more effective than arm 0 when comparing CD4 T-cell count over time (cf. original hazard ratio: HR = 0.49 (95% CI, 0.39–0.63); *p* = 1.22e-08 vs. avatar data: HR = 0.40 (95% CI, 0.31–0.52); *p* = 1.47e-11) (see Supplementary Table 1 for additional comparative statistics). For the WBCD dataset, Fig. 1d shows the *F*-score comparison for each cancer prediction variable. *F*-score computations for the avatar (green) and original (orange) datasets were similar. The predictive models selected the same variables, yielding the same feature importance. These models have comparable prediction performances (original: *AUC*=99.46 (std = 0.25) vs. avatar: AUC = 99.84 (std = 0.12); see Supplementary Table 2 for additional predictive statistics). Overall, these results suggest that avatar data support similar analyses with potentially decreased variance.

### Comparison of the Avatar method to other synthetic data generation methods

**Fig. 2:**
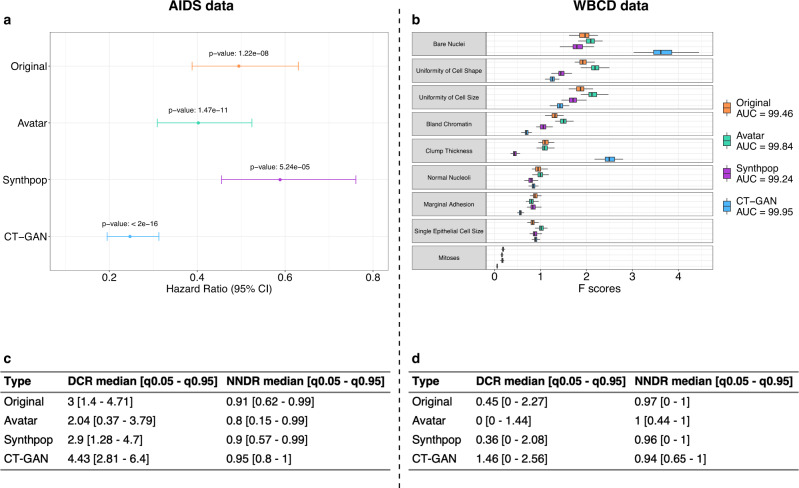
Comparative results of utility and privacy for original avatar datasets, Synthpop, and CT-GAN data. **a** Hazard ratio between arm 0 and arm 1 per synthetic data generation method comparison (Avatar method: green, Synthpop: purple, CT-GAN: blue) with original reference (orange). Error bars represent the 95% confidence interval. **b** Boxplot comparison of *F*-scores obtained in SVM models per variable and per synthetic data generation method (Avatar ethod: green, Synthpop: purple, CT-GAN: blue) over 100 iterations with original reference (orange). Boxplots present the median, first, and third quartiles. Minimum whisker equals (Q1–1.5*IQR) and maximum equals (Q3 + 1.5*IQR). **c**, **d** Summary table (**c**) for AIDS and (**d**) for WBCD, of DCR and NNDR median values and quantiles (0.05–0.95) for the three synthetic data generation methods. Original is obtained by applying both metrics on original 70% sampling and 30% holdout original data. AUC area under the ROC curve, CI confidence interval, Q1 first quartile, Q3 third quartile, IQR interquartile range, DCR distance to the closest record, NNDR nearest neighbor distance ratio, q0.05 5th percentile, q0.95 95th percentile, SVM support vector machine, CT-GAN conditional tabular generative adversarial network.

After showing that the Avatar method could reproduce the original analyses, we evaluated its performance compared with two other synthetic data generation methods (Synthpop and CT-GAN). Figure 2 presents the main statistics of the comparative analysis (see Supplementary Figs. 1 and 2 for method-specific results). Figure 2a displays the hazard ratio obtained with the original data and the three synthetic data generation methods on the AIDS analysis. The three synthetic datasets lead to the same conclusions as the original data: arm 1 is more effective than arm 0 when comparing CD4 T-cell count over time (Wald test—Original *p*-value: 1.22*e*-08, avatar *p*-value: 1.47*e*-11, Synthpop *p*-value: 5.24*e*-05, CT-GAN *p*-value: <2*e*-16). The Hazard ratio values obtained with the avatar and Synthpop AIDS data are within the confidence interval of the original data. The data produced by CT-GAN induce an underestimation of the hazard ratio. Figure 2b compares the AUC and the *F*-scores of each variable obtained for the original WBCD data and its three synthetic versions. The SVM models resulting from original and synthetic data have comparable prediction performances for WBCD. (Original AUC: 99.46, avatar AUC: 99.84, Synthpop AUC: 99.24, CT-GAN AUC: 99.95). The *F*-scores obtained with the avatar data are the closest to the original *F*-scores. The higher *F*-scores obtained with CT-GAN data for the *Bare Nuclei* and *Clump Thickness* variables indicate that the model introduces bias giving more importance to these two variables in predicting outcome. Overall, the 3 synthetic datasets lead to the same conclusion as the original data for each use case.

Beyond the preservation of the statistical utility, the main goal of any anonymization method is to prevent re-identification. We compared the distance to closest record^28^ (DCR) and nearest neighbors distance ratio^28,36^ (NNDR) median values obtained with original data and the three synthetic datasets for each use case. Figure 2c, d present privacy results for AIDS and WBCD. Median DCR for original data is 3 for AIDS and 0.45 for WBCD. CT-GAN data is the furthest from the original data for both use cases with a median DCR of 4.43 for AIDS and 1.46 for WBCD. In comparison, Synthpop data offer a median DCR of 2.9 for AIDS and 0.36 for WBCD, and avatar data a median DCR of 2.04 for AIDS and 0 for WBCD. In both use cases, the median DCR of the CT-GAN data is higher than the original reference. It indicates that the CT-GAN data is on average more distant from the training data than the holdout of the original data itself. In comparison, the data generated by Synthpop are at an equivalent distance to the holdout original data and the avatar data are closer. The DCR metric states that CT-GAN and Synthpop data provide more privacy than avatar data. Regarding NNDR, original data yields a ratio of 0.91 for AIDS and 0.97 for WBCD. The three synthetic methods show similar results with 0.8 (AIDS), 1 (WBCD) for avatar data, 0.9 (AIDS), 0.96 (WBCD) for Synthpop, and 0.95 (AIDS), 0.94 (WBCD) for CT-GAN. The high NNDR values for all methods (0.8, 0.9, 0.95, respectively, for AIDS and 1, 0.96, 0.94, respectively, for WBCD) indicate that none of the methods generate close and isolated original and synthetic pairs. In terms of privacy, the three datasets present satisfactory (≥0.8) NNDR results, however, these metrics alone do not allow us to rule on the anonymous nature of the data.

### Avatar method and assessment of the re-identification risk with patient-centric metric

**Fig. 3:**
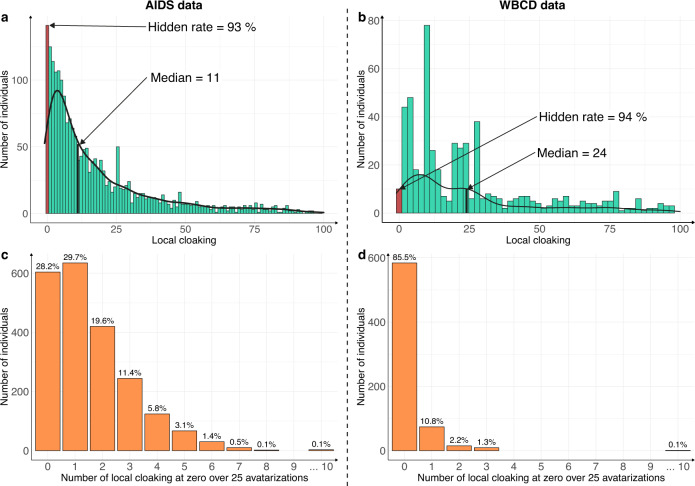
Quantification of re-identification risk of sensitive data using the avatar dataset. The risk of re-identifying an individual in the avatar dataset is near zero. **a**, **b** Distribution of the local cloaking for **a** AIDS (hidden rate: 93%, median = 11) and **b** WBCD (hidden rate: 94%, median = 24). **c**, **d** show the histograms of individuals according to the number of times they had a local cloaking of zero for the **c** AIDS and **d** WBCD datasets. In both cases, the experiment was conducted on 25 independent avatar simulations (*k* = 20).

The patient-centric nature of the Avatar method allows the computation of supplemental-specific metrics not applicable to synthetic data generation methods based on the training of a global model. Figure 3 shows the distribution of the local cloaking metric (3a: AIDS dataset; 3b: WBCD dataset). In panel 3a, the median local cloaking of 11 shows that there is a median of 11 avatar simulations between an original observation of the AIDS dataset and its simulated avatar. The hidden rate of 93% means that 7% of the individuals produced the avatar that most resembled them (local cloaking equal to 0). In panel 3b, the median local cloaking was 24, indicating that there is a median of 24 avatar simulations between the original WBCD dataset observations and their avatar simulation. The hidden rate was 94%, suggesting strong data protection. In both the AIDS and WBCD datasets, less than 7% of individuals appeared to be unprotected because their avatar simulations showed a local cloaking of 0. Figure 3c, d present the number of times each sensitive individual observation generated the avatar simulation closest to them over the 25 independent avatarizations. Individuals (AIDS: 28.2% vs. WBCD: 85.5%) do not generally get local cloakings of 0. For the AIDS dataset, only three individuals (0.1% of the dataset) had 10 times or more a local cloaking of 0 after 25 avatarizations. For the WBCD dataset, one individual (0.1% of the dataset) had 10 times or more a local cloaking of 0 after 25 avatarizations (see Supplementary Fig. 3 for additional details). Overall, these metrics for the Avatar method demonstrate that the re-identification risk is quantifiable and provides protection for every single data contributor. Additionally, according to Fig. 3c, d, the generation of an avatar simulation that resembles the original individual occurs at random and is beyond the attacker’s knowledge.

### Impact of local model size on avatar generation

**Fig. 4:**
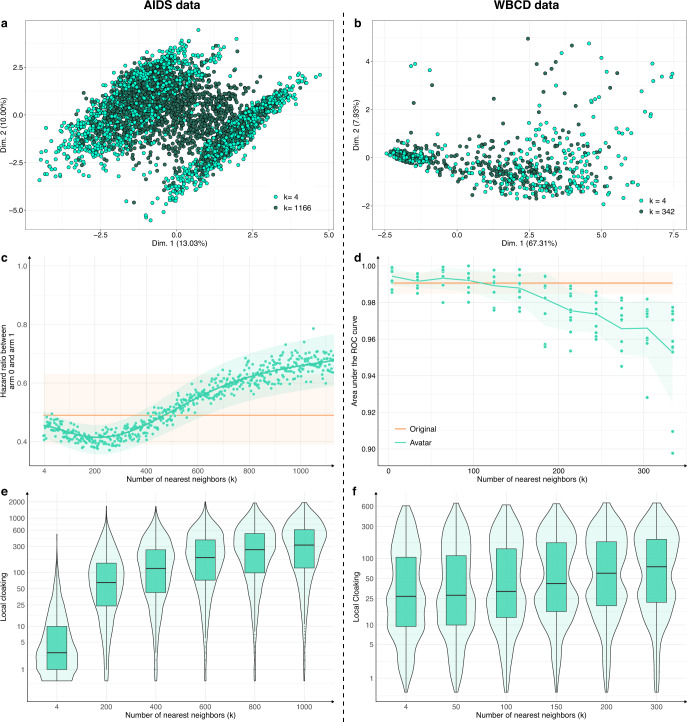
Influence of *k* on statistical relevance and re-identification risk. High *k* values lower the preservation of statistical information of the dataset while enhancing privacy: **a**, **b** FAMD projections of **a** two AIDS avatar simulations with *k* = 4 (light green dots) and *k* = 1166 (dark green dots) and **b** two WBCD avatar simulations with *k* = 4 (light green dots) and *k* = 342 (dark green dots) in their original data FAMD projection space. Contrary to Fig. 1 a, b, Fig. 4 a, b only present avatar data. **c** Hazard ratio evolution for arm 1 compared with arm 0 as a function of *k*. The green zone represents the 95% CI of the hazard ratio mean. The orange line represents the original data results. **d** Accuracy evolution as a function of *k*. For each *k*, 10 train/test datasets (70/30) SVM models were computed. Green zones represent 95% CI. Orange lines and associated areas represent the original data AUC mean and associated 95% CI. A high *k* influence on data privacy. **e**, **f** Comparison of the local cloaking distribution (base-10 log scale) for low *k* to high *k*. Boxplots present the median, first, and third quartiles. FAMD factor analysis for mixed data, AUC area under the ROC curve, SVM support vector machine, CI confidence interval.

The number of neighbors *k* is a crucial parameter. For each use case, Fig. 4a, b compare the FAMD projections of avatar simulations generated with a low *k* (respectively, 0.2% and 6% of the total number of individuals, light green) and avatar simulations generated with a high *k* (respectively, 50%–55% of the total number of individuals, dark green). The dataset structures were well-conserved for the lowest *k* values (compared with Fig. 1a, b; *k* = 20). The structures and their boundaries faded with the highest value of *k*. Figure 4c, d show the evolution of the major endpoint estimations as a function of *k* (hazard ratio for AIDS; AUC of cancer prediction for WBCD). The estimations stayed within the confidence interval of the estimations from the original dataset when *k* was between 4 and 750 for the AIDS dataset and between 4 and 150 for the WBCD dataset. For low *k* values, the effect size tended to be overestimated; for higher *k* values, the effect size tended to be underestimated.

Figure 4e, f present the local cloaking distributions according to *k*. Lower *k* values indicated denser and lower local cloaking distributions. The median local cloaking increased accordingly with *k*. Overall, higher *k* values indicated less conserved data structure and margins and more deviated estimations. However, higher *k* values indicated more protected individuals. The statistical relevance remained valuable when *k* reached its highest value.

## Discussion

Herein, we present and evaluate a new method, the Avatar method, to generate synthetic data. We replicate this approach with two other synthetic datasets generated using different methods (Synthpop, CT-GAN) and compare utility and privacy results. These methods aim to protect sensitive data from re-identification while retaining the statistical value of the dataset. We use a publicly available clinical trial dataset comparing HIV treatments and a breast cancer prediction dataset for privacy and utility retention assessments. Evaluation of the method is achieved by comparing results obtained from sensitive data with those obtained from avatar data and result in the same interpretation for both datasets. All synthetic data show comparable utility with the original data with an accountable level of privacy. The Avatar method is patient-centric (i.e., it uses the characteristics of a single patient as the starting point of its statistical modeling). Even if each individual is at the origin of the creation of their avatar simulation, they do not directly contribute to the local modeling of their Avatar generation. This seemingly paradoxical nature of the method limits re-identification risks. The choice to generate each avatar randomly within a local space differs from the desire to maximize the distance between the original and generated individuals. This specificity implies that the generated avatar simulations could potentially be closer to the original data in denser areas than those generated with other methods. The method is based on multidimensional projections and a selection of local neighbors in a reduced space. In this manuscript, we use FAMD^37^ to project the individuals in an Euclidean space. In practice, other methods, such as discriminant analysis^38^, t-SNE^39^, and autoencoders^40^ could be considered. The use of multivariate analysis avoids the curse of dimensionality by searching for neighbors in a reduced space, optimizing the core computation (see Supplementary Table 3 for comparison of computation times) of the KNN at the same time. Compared with other methods, the projection of the individuals in a mathematically explainable space allows one to understand the influence of variables on the neighbor computation. The choice of the projection method is a balance between computational requirements and the relevance of mathematical modeling, including distance choice, potential loss of information, and noise propagation. It underlines the central role of the projection used in Avatar method. The limitations of the Avatar method are related to the limitations of the projection method it uses^41^. Multiple data projections or transformations can be used (if any), for example, the use of Fourier transform to handle time series instead of tabular data is presented in another context^42^.

The parameter *k* has a strong influence on data privacy and quality and needs to be adapted given the data’s sensitivity and intended use. We show that a worthy level of protection can be achieved even with a low *k* value. This parameter is currently uniformly applied to each simulation; future work may propose a dynamic adaptation of *k* depending on the records surrounding density. However, while the synthesized individuals reflect the variability and quality of the original data, synthetic data generation methods allow to generate a synthetic cohort of infinite (lower or greater) size. For example, this method can be used to compute empirical distributions of any estimates from the original dataset (see Supplementary Fig. 4 for the hazard ratio and Supplementary Fig. 5 for the *F*-score).

The Avatar method preserves the structure of the original dataset and reaches high signal retention. The comparison of the Avatar method with Synthpop and CT-GAN shows that the performance of signal retention is similar or greater with avatar simulations for the dataset treated in this experiment. Using the default parameters, the Avatar method produces data that more resemble the original data than the Synthpop and CT-GAN data. The lower DCR values observed with the methods on the WBCD use case are related to the reduced variance of the dataset and the presence of duplicates. The high NNDR value (≥0.8) observed for all methods on both use cases indicates that none of the methods has the particularity to generate close and isolated original and synthetic pairs. In addition, the patient-centric nature of the Avatar method enables the calculation of additional privacy metrics. The *local cloaking* and *hidden rate* metrics account for privacy at the individual level. By comparing the Avatar method with Synthpop and CT-GAN, we illustrate that the choice of a synthetic data generation method is always a balance between utility and privacy. While synthetic data generation opens the possibility of multiple secondary uses, in particular for open-data applications, it is influenced by the primary data usage context. The adoption of methods is driven by the possibility to fine-tune this balance. Where other data simulation methods (e.g., GAN^25–28^) use a global model to mimic the overall original statistical properties, the Avatar method uses local simulation that facilitates the computation of privacy protection metrics that satisfy EDPB^16^. Explainability and accountability are also reinforced at both global and local levels, while parameter tuning enables exploration of the method behavior, which may be seen as particularly crucial in health applications^43^. The Avatar method was built aiming for interpretability at each step for both privacy protection and signal retention. The possibility to assess and adapt the privacy level to data sensitivity and their context of use led the French data protection authority (CNIL) to consider the Avatar method as compliant with anonymization in the sense of GDPR. Comparing the two families of anonymization techniques^16^: randomization (e.g., noise addition, permutation, and differential privacy^21^) and generalization (e.g., K-anonymity^19^, L-diversity^20^, and T-closeness^44^), synthetic data generation methods allow high signal conservation^33^ while allowing privacy evaluation. These methods are compatible with the use of randomization and generalization methods and can be combined with them in a treatment depending on the intended use of the data. Compared to differential privacy, synthetic data generation methods such as the Avatar method have more flexibility in their use^24^ but do not have an a priori mathematical proof of the privacy level provided. A current evolution of the method could deliver a local model that would be differentially private. The control of the level of utility and privacy allows for adapting the optimal treatment to the use, particularly in the field of health where keeping utility is essential although the data are sensitive.

Synthetic data are becoming a key tool in an open-data world and are streamlining making data available to data scientists^45^. With the avatar dataset, researchers do not need to expose sensitive patients or risk patients’ privacy when publishing their results. This should be a proposed standard in analyzing biomedical data and data in general and has already proven its relevance to promote reproducibility^46^. We develop our analysis in the specific case of tabular data, but other real-life data sources offer multiple possibilities, such as images, high-dimensional data (-omics data), tracking data, geospatial data, or time series. Applying the Avatar method to these specific data types will require specific developments.

Synthetic data generation methods promote collective intelligence and enable sharing codes that apply seamlessly to both original and synthetic data^33,46^. The use of synthetic data allows unleashing personal data potential to improve future healthcare systems while ensuring individual privacy. The Avatar method respects GDPR constraints by enabling data sharing without compromising privacy. Personal data should be restricted to personal use. Not using synthetic data when possible, undermines the trust required to build an open-knowledge society.

## Methods

### Description of the Avatar method

**Fig. 5:**
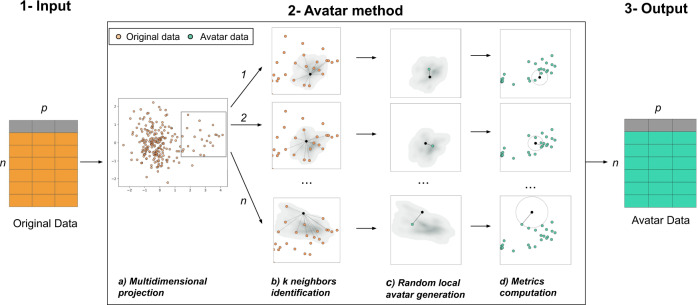
The Avatar method uses local modeling to stochastically generate a synthetic individual, termed an avatar simulation. **(1)** Original pseudonymized sensitive data. **(2)** The core of the Avatar method consists of four steps: (a) individuals are projected in a multidimensional space; (b) pairwise distances are computed to find the *k* nearest neighbors (here *k* = 12) in a reduced space; (c) a synthetic individual is pseudo-randomly generated in the subspace defined by the neighbors; (d) privacy metrics are evaluated. **(3)** Output of the dataset of synthetic data. More details are provided online (https://docs.octopize.io/).

The Avatar method uses a patient-centered approach. Each original observation generates a local random simulation leading to its avatar simulation. We consider a pseudonymized sensitive dataset of size *n* × *p*, where *n* rows represent individuals, and *p* columns are variables. Variables can either be continuous variables, categorical variables, booleans or dates. The Avatar method aims to create a new dataset of *n* synthetic observations and *p* variables with consistent yet different values compared with those of the original dataset. Avatar data are a synthetic dataset composed of mathematically simulated individuals, originating from the original sensitive dataset. Figure 5 illustrates this operation. In short, the core of the Avatar method has three major steps. (1) Input: the input data are a pseudonymized tabular dataset. (2) The core of the Avatar method: (2a) individual observations are projected in a complete multidimensional space using factor analysis technique (e.g., PCA^47^, FAMD^37^, and MCA^48^). (2b) Using the first number of dimensions (*nd)* of this space, pairwise distances are computed between each sensitive individual observation to find the *k* nearest neighbors with the KNN algorithm^49,50^, which define a local area. (2c) For each individual, a single avatar simulation is pseudo-stochastically drawn in its local area. Considering an individual *O* in the original dataset *D*, the aim is to create an avatar simulation *A* for each *O*. Once the *k* neighbors of *O* are identified, a random weight is affected for each neighbor^51^. In this study, those *k* weights are defined as follows:

1 $$For\,i\,in\left[ {1,..,{{{\mathrm{k}}}}} \right],P_i = D_i \times R_i \times C_i$$

with:

- *D*_*i*_ the inverse of the distance between *O* and its *i*^th^ neighbor *k*,
- *R*_*i*_ ~ ξ(1): a random weight following an exponential distribution, with *λ* = 1,
- $C_i = (\frac{1}{2})^j$: a contribution, where *j* is the value at *i*^th^ index of the randomly shuffled vector [1, 2,…, *k*].

For example, considering an individual O having *k* = 2 neighbors distant from 3 and 5 in the Euclidean space and with the randomly shuffled vector [2,1],

$$P_1 = 1/3 \times {\mathrm{random}}\_{\mathrm{value}} \times 1/4$$

$$P_2 = 1/5 \times {\mathrm{random}}\_{\mathrm{value}} \times 1/2$$

Finally, each weighting term is divided by the sum of all the neighbor’s weights as follows:

2 $$W_i = \frac{{P_i}}{{\mathop {\sum }\nolimits_{j = 1}^k P_j}}$$

where *W*_*i*_ is the weight of the *i*^th^ nearest neighbor.

Each of the *k* nearest neighbors of the individual *O* yield a weight *W*_*k*_ between 0 and 1. Avatar simulation coordinates are then generated at the weighted center of the *k* nearest neighbors. The parameters *k*, *nd*, and others, such as variable weights, drive the randomness and information content of the simulations. (2d) The properties of the avatar dataset are evaluated by computing both Avatar-specific privacy metrics and signal retention metrics^24^. Importantly, this step allows reiterating phase 2b if the metrics lack sufficient privacy or acceptable statistical conservation. (3) Output: the avatar simulations are reverse transformed from their coordinates in the full modeling space into values of the initial structured dataset by performing the reverse mathematical process of the factor analysis used. Synthetic observations (rows) are shuffled to remove the link between the original individuals and the avatar simulations.

The method is controlled by two types of parameters: (1) parameters affecting the local environment: distance used (e.g., Euclidean or Mahalanobis^52^), number of neighbors (*k*), parameters of multidimensional projection (e.g., standardization, number of dimensions used in neighbor identification and variables custom weighting for projection) and (2) parameters affecting how stochastic an avatar generation can be: the weights distribution law over neighbors (equal or unbalanced contribution) and the percentage of perturbation applied to the avatar for each variable.

### Privacy metrics definition

After generating the synthetic dataset, metrics are required to assess privacy. For each dataset and each method, we computed two metrics used in the literature to evaluate the privacy of any synthetic data: the distance to closest record (DCR^28^) and the nearest neighbor distance ratio (NNDR^28,36^). The DCR is defined as the Euclidean distance between each synthetic record and its closest corresponding real neighbor. The higher this distance, the better the privacy level. The NNDR is the ratio between the Euclidean distance of the closest and the distance of the second closest real neighbor for each synthetic record (see Supplementary Fig. 6 for details). The NNDR is bounded in [0, 1], the higher the better the privacy level. Of the three EDPB criteria^16^, singling out represents the most unfavorable and sensitive attack scenario. Herein, we also introduce two metrics specific to the Avatar method addressing the singling-out issue, *local cloaking* and *hidden rate*. It leverages the local nature of the model used to sample each avatar simulation. We place ourselves in the membership attack scenario^36,53^. The attacker seeks to determine an individual’s membership in a cohort by establishing a link between a sensitive individual and an avatar simulation. In this context, the most likely attack is a distance-based linkage attack^53^. For each sensitive individual, the local cloaking metric counts the number of avatar simulations that are more similar (i.e., closer in the multidimensional space) to the original data than the one avatar produced from the data. The hidden rate metric represents the percentage of individuals in the original dataset whose avatar simulation is not the most similar to them. This metric evaluates the probability of an attack being wrong when it associates an avatar simulation with the individual to whom the avatar simulation is most similar (see Supplementary Fig. 7 for details). Higher values for both metrics imply a better privacy level.

### Application of the method to biomedical dataset 1: Acquired Immunodeficiency Syndrome (AIDS) clinical trial

The AIDS dataset includes 2139 patients and 26 variables for HIV-infected patients who participated in a clinical trial published in 1996 in the *New England Journal of Medicine*. The clinical trial had four arms and was analyzed by Hammer et al.^54^. The main endpoints used were survival and a 50% drop in CD4+ cell counts.

### Application of the method to biomedical dataset 2: Wisconsin Breast Cancer Diagnosis (WBCD) prediction issue

The WBCD dataset comprises 683 observations and 10 variables. It is frequently used for student training purposes and can be downloaded from the University of California Irvine machine-learning repository^55^. The outcome corresponds to the tumor severity: benign (*n* = 444) vs. malignant (*n* = 239). The other nine features are built from imaging-specific annotations and are graduated from 1 to 10. Feature selection (*F*-score computation) and a support vector machine (SVM) were used to predict the severity of a patient’s breast cancer diagnosis as per Akay et al.^56^.

### Protocol

For each use case (AIDS and WBCD), synthetic datasets were generated. We generated a synthetic dataset using the Avatar method^57^ with the parameter *k* = 20. To evaluate the ability to retain the utility of the original datasets, we performed four analyses (two per use case). For both AIDS and WBCD, we compared the multidimensional reduction representation of each original dataset with its synthetic avatar version. For AIDS we compared the survival curve of two treatments and the hazard ratio value computed with original and avatar data. For WBCD, we compared the *F*-score computation (see supplementary method 1 for details) and classification performance (area under the receiver operating characteristic curve; AUC, see supplementary method 2 for details) of the original and avatar data. We then evaluated the performance of the Avatar method against two other synthetic data generation methods. For both use cases, we generated two additional synthetic datasets, one relying on classification and regression tree (Synthpop^30^), and the second one using conditional generative adversarial network (CT-GAN^27^). To compare methods on the utility preservation ground, the two additional synthetic datasets for each use case went through the same pipeline of analysis described above. For privacy comparison of the synthetic data generation methods, we used DCR and NNDR metrics in both use cases. For this analysis, we generated one synthetic dataset per method (Avatar, Synthpop, CT-GAN) based on 10 sampling of 70% of the original set, i.e., 10 synthetic datasets per method per use case. The DCR and NNDR were computed between the generated synthetic data and the original sampling. We also computed DCR and NNDR between sampling and the holdout 30% original data to be used as a comparison basis. Since the Avatar method has the particularity of being patient-centric, we were able to compute the specific re-identification metrics (local cloaking and hidden rate) on avatar data. The last part of the study focuses on evaluating the behavior of the Avatar method. To illustrate the stochasticity of the method, we performed 25 Avatar generation experiments (*k* = 20) of each dataset, and for each individual, we looked at the number of times a distance-based linkage attack would have led to correct re-identification. Then, to evaluate the impact of *k* on AIDS and WBCD data, we performed survival analyses over 10 Avatar generation expriments for each *k* ranging from 4 to 1200 (to achieve half-size of the dataset) for AIDS and we computed the AUC over 10 Avatar generation experiments for multiple *k* ranging from 4 to 334 (to exceed the size of the smallest group of interest, i.e., 239 malignant tumors) for WBCD.

## Supplementary information

Supplementary material

## Data Availability

The reference datasets (AIDS and WBCD) and all synthetic datasets used in this study as well as data that support the findings of this study have been deposited in the public “avatar-paper” repository available on GitHub (https://github.com/octopize/avatar-paper/tree/main/datasets).

## Code availability

To promote transparency and reproducibility of the results, all synthetization parameters used for Avatar, Synthpop and CT-GAN, and analysis codes are available on GitHub (https://github.com/octopize/avatar_paper) without any restriction access. Analyses were performed using R (version 4.1) and Python (version 3.9).
