# Supplementary material for: Patient-centric synthetic data generation, no reason to risk re-identification in biomedical data analysis
Source: NPJ Digit Med. 2023 Mar 10;6:37. doi: 10.1038/s41746-023-00771-5 (PMC10006164; doi:10.1038/s41746-023-00771-5)
Supplement: Supplementary file 1 — Supplementary material [file 41746_2023_771_MOESM1_ESM.pdf]

SUPPLEMENTARY TABLE 1

Supplementary Table 1 compares the hazard ratios between the avatar and its original data. Statistically significant hazard ratios for the original data remained significant with the avatars. Furthermore, the hazard ratio results for the avatar were in the 95% confidence interval range of the original data. Therefore, no significant differences occurred between using the avatar data and the original data. We conclude with the same results.

Table 1. Comparison of the relative hazard ratio results obtained with the original and avatar data. In each case, the reference group is arm 0 (p-values refer to Wald test).

	Hazard Ratio	95% Confidence Interval	*p*-value
Original arm 1	0.49	0.39–0.63	9.19e-09
Avatar arm 1	0.40	0.31–0.52	9.02e-12
Original arm 2	0.52	0.41–0.67	1.07e-07
Avatar arm 2	0.48	0.37–0.62	8.68e-09
Original arm 3	0.59	0.47–0.73	3.68e-06
Avatar arm 3	0.53	0.42–0.67	1.04e-07

SUPPLEMENTARY TABLE 2

For the WBCD datasets, we trained SVM models (70% training, 30% tests) with five features according to F-score results. Supplementary Table 2 compares the classification performances for the best original and avatar models. We obtained the same classification performance for both the original and avatar datasets.

Table 2. Evaluation of classification performances, accuracy, specificity, sensitivity, PPV (positive predictive value), NPV (negative predictive value), and AUC (area under the receiver operating characteristics curve), with standard deviation for each best model.

	Avatar	Original
Accuracy	97.04 (± 0.99)	96.58 (± 1.25)
AUC	99.84 (± 0.12)	99.46 (± 0.25)
NPV	96.07 (± 2.77)	96.08 (± 2.88)
PPV	97.60 (± 1.62)	96.85 (± 1.35)
Sensitivity	97.87 (± 1.49)	97.88 (± 1.51)
Specificity	95.69 (± 2.83)	94.35 (± 2.28)

SUPPLEMENTARY TABLE 3

Table 3. Comparison of computational time required by the three synthetic data generation methods (Avatar, Synthpop, CT-GAN) to generate a synthetic cohort of AIDS and WBCD. The value displayed is the mean time in seconds over 10 iterations with the corresponding standard deviation.

	AIDS	WBCD
Avatar	0.58 s (± 0.03)	0.15 s (± 0.03)
Synthpop	2.42 s (± 0.26)	0.17 s (± 0.04)
CT-GAN	279.74 s (± 9.14)	79.69 s (± 3.02)

SUPPLEMENTARY FIGURE 1

The Synthpop synthetic data generation method retained the statistical value of the datasets. For the AIDS (Figure 1a) and WBCD (Figure 1b) datasets, the factor analysis of mixed data (FAMD) projection of the first two components showed that the original and Synthpop data overlapped, including the outliers. This result indicates the preservation of the structural information. Figure 1c compares the survival curves calculated with the Synthpop dataset and the original AIDS dataset. In both treatment arms, the survival curves of the Synthpop (dotted line) and originals (continuous line) follow the same trend. Regarding the survival curves, the analysis of the Synthpop data is leading to the same interpretations as the one obtained with sensitive data. The statistical p-value are computed using Wald test. The main trial results remained unchanged: arm 1 was more effective than arm 0 when comparing CD4 T-cell count over time (cf. original data: $HR = 0.49\,(95\%\,CI, 0.39 - 0.63)$; $p = 9.19e - 09$ vs Synthpop data: $HR = 0.59\,(95\%\,CI,\ 0.46 - 0.76)$; $p = 5.24e - 05$). For the WBCD dataset, Figure 1d shows the F-score comparison for each cancer prediction variable. F-score computations for the Synthpop (purple) and original (orange) datasets were similar. Regarding the F-scores, the predictive model selected approaching variables, yielding comparable feature importance. These models have similar prediction performances (original: $AUC = 99.46\,(std = 0.25)$ vs Synthpop: $AUC = 99.24\,(std = 0.17)$. Overall, these results suggest that Synthpop data support similar analyses. The Synthpop synthetic data lead to the same interpretations as those obtained with pseudonymized data.

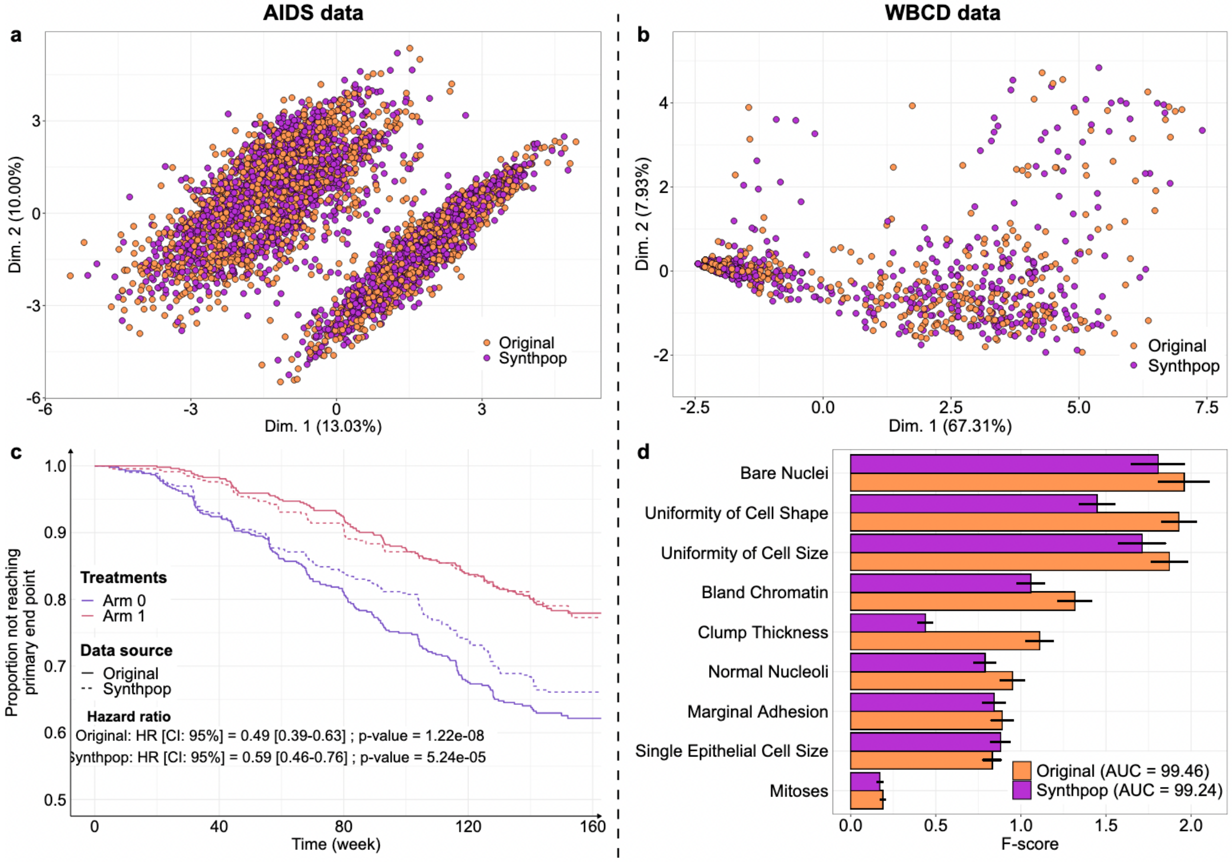

Figure 1: Comparative results of analyses based on original and Synthpop data

Legend: (a, b) FAMD projections of the (a) AIDS and (b) WBCD synthetic Synthpop data in the original data space (original data in orange, Synthpop in purple). Synthpop and original data are overlaid and share the same space built from the original observations. (c) Distributions of times to events were estimated with the method of Kaplan and Meier and compared with the log-rank test and Cox proportional-hazards model, with a comparison between the original (plain lines) and AIDS Synthpop (dotted lines) for arms 0 (purple lines) and 1(red lines). The statistical p-values are computed using Wald test. The original and Synthpop WBCD datasets were separated into 70 training trials and 30 tests (100 times). (d) Comparison of F-scores for each variable. Error bars represent the 95% confidence interval. SVM machine-learning models were performed using five features selected by F-score. The AUC is presented for the original and Synthpop datasets. Abbreviations: FAMD: factor analysis for mixed data; AUC: area under the ROC curve; SVM: support vector machine, CI: confidence interval.

SUPPLEMENTARY FIGURE 2

The CT-GAN synthetic data generation method retained the statistical value of the datasets. . For the AIDS (Figure 2a) and WBCD (Figure 2b) datasets, the factor analysis of mixed data (FAMD) projection of the two first components showed that the original data and CT-GAN have a decent overlap. We note however that a significant number of synthetic individuals have been generated between the two clusters distinguishable in the projection. Figure 2c compares the survival curves calculated with the CT-GAN dataset and the original AIDS dataset. As for original survival curves (continuous line), CT-GAN (dotted line) survival curve for arm 1 shows a higher proportion of patients not reaching the primary endpoint

over time than arm 0 survival curve. The analysis of the CT-GAN data is leading to the same interpretations as the one obtained with sensitive data. The statistical p-value are computed using Wald test. The main trial results remained unchanged: arm 1 was more effective than arm 0 when comparing CD4 T-cell count over time (cf. original data: $HR = 0.49\,(95\%\,CI, 0.39 - 0.63)$; $p = 9.19e - 09$ vs CT-GAN data: $HR = 0.25\,(95\%\,CI,\ 0.20 - 0.31)$; $< 2e - 16$). We note however that trends from CT-GAN survival curves differ from originals by not adopting asymptotic characteristics at the end. For the WBCD dataset, Figure 2d shows the F-score comparison for each cancer prediction variable. F-score computations for the CT-GAN (blue) and original (orange) show different behaviors. The model based on CT-GAN data brings significantly more importance to the variables *Bare Nuclei* and *Clump Thickness*. For the rest, the results are comparable. These models have similar prediction performances (original: $AUC = 99.46\,(std = 0.25)$ vs CT-GAN: $AUC = 99.95\,(std = 0.13)$. Overall, these results suggest that CT-GAN data support similar analyses. The CT-GAN synthetic data lead to interpretations approaching those obtained with pseudonymized data.

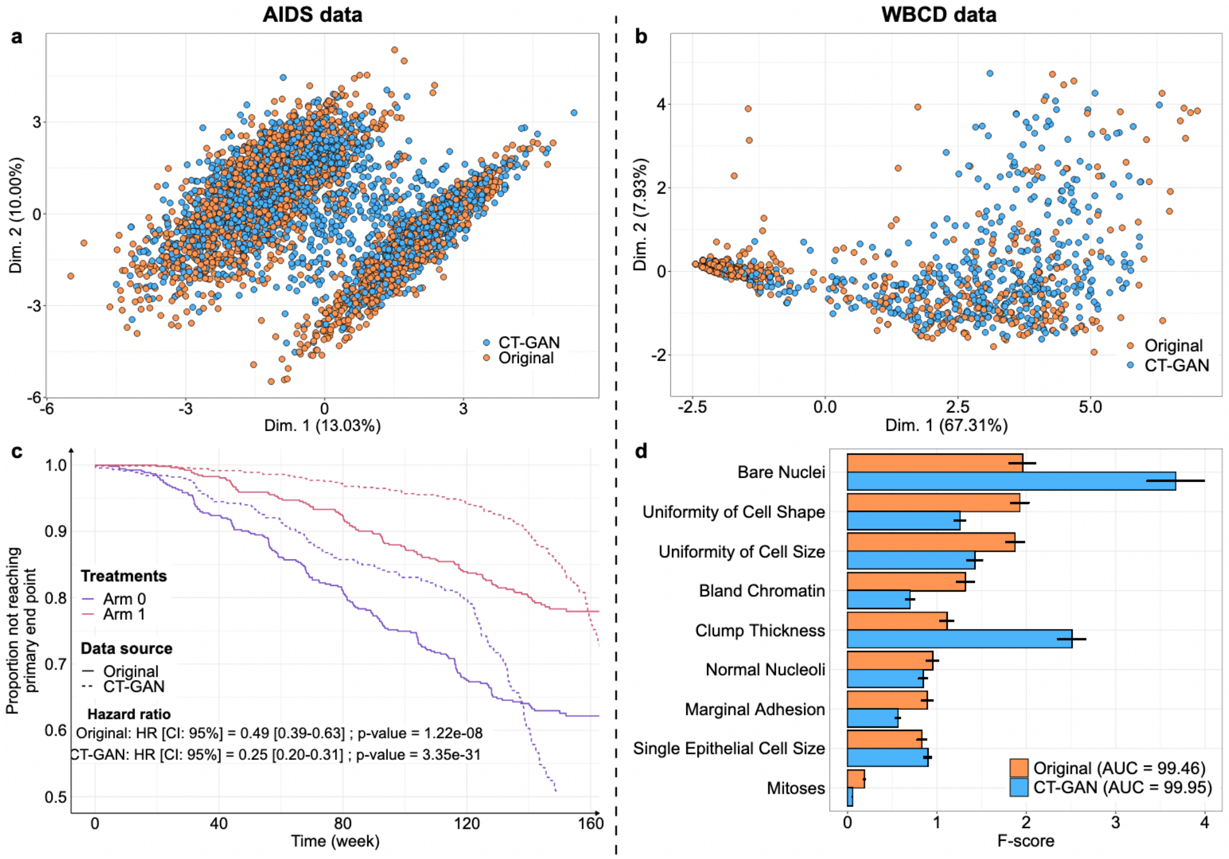

Figure 2: Comparative results of analyses based on original and CT-GAN data

Legend: (a, b) FAMD projections of the (a) AIDS and (b) WBCD synthetic CT-GAN data in the original data space (original data in orange, CT-GAN in blue). CT-GAN and original data are overlaid and share the same space built from the original observations. (c) Distributions of times to events were estimated with the method of Kaplan and Meier and compared with the log-rank test and Cox proportional-hazards model, with a comparison between the original (plain lines) and AIDS CT-GAN (dotted lines) for arms 0 (purple lines) and 1(red lines). The statistical p-values are computed using Wald test. The original and CT-GAN WBCD datasets were separated into 70 training trials and 30 tests (100 times). (d) Comparison of F-scores for each variable. Error bars represent the 95%

confidence interval. SVM machine-learning models were performed using five features selected by F-score. The AUC is presented for the original and CT-GAN datasets. Abbreviations: FAMD: factor analysis for mixed data; AUC: area under the ROC curve; SVM: support vector machine, CI: confidence interval, CT-GAN: conditional tabular generative adversarial network.

SUPPLEMENTARY FIGURE 3

Supplementary Figure 3 shows the conditional probability for an individual to get a local cloaking score regarding the previous.

The smallest conditional probabilities are evenly distributed in the first column. It shows that it is unlikely to yield two successive avatarizations with a local cloaking of zero.
The avatarizations are considered independent if the probability of a local cloaking of zero equals the conditional probability of a local cloaking of zero in a second avatarization, knowing that the first avatarization had a local cloaking of zero, i.e. $P(X = 0) = P(X_2 = 0|X_1 = 0)$

Using 25 AIDS dataset avatarizations yields

$$P(X = 0) = 0.06 \text{ and } P(X_2 = 0|X_1 = 0) = 0.09$$

These two probabilities are both low and close; thus, the avatarization can be interpreted as quasi-independent from one simulation to the next. A distance attack leading to a correct re-identification is unrelated to the value of the individual's data but mostly related to the stochasticity of the method.

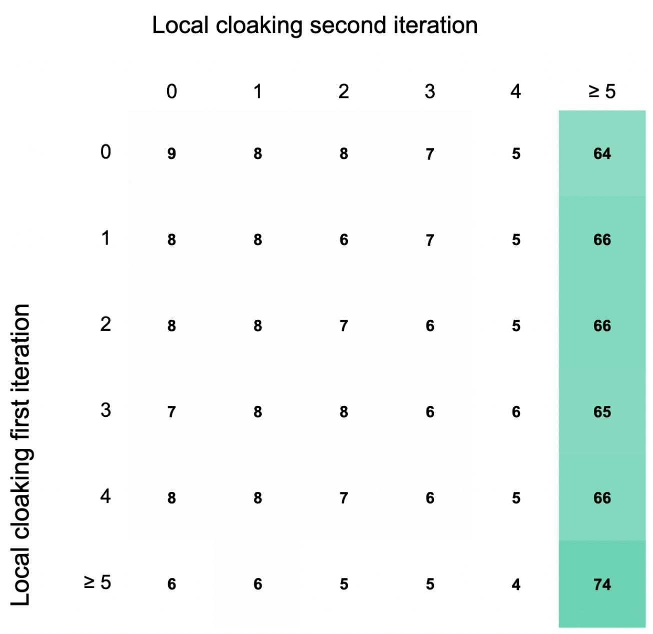

Figure 3: Heatmap of conditional probabilities (AIDS dataset).

Legend: Each case represents the proportion (in percent) of individuals with a local cloaking at {0, 1, 2, 3, 4 or 5 and more} at a second iteration knowing they had a local cloaking at {0, 1, 2, 3, 4 or 5 and more} at a first iteration.

SUPPLEMENTARY FIGURE 4

We performed 100 avatarizations of the AIDS datasets with the same parameters (*k*=20). For this dataset, we focused on the hazard ratio distribution for arms 1, 2, and 3 compared to arm 0. Results obtained with original data are then compared to those obtained with avatar data. We obtained a significant effect of the three treatments compared to arm 0. Arm 1 is showing the best performance.

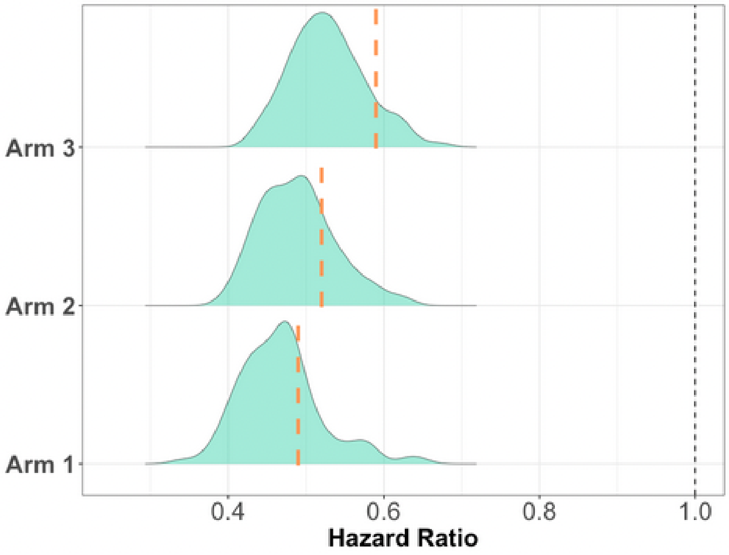

Figure 4: Hazard ratio distributions. 100 independent avatarizations of the AIDS dataset were performed (*k* =20). The distributions of the hazard ratio for arms 1, 2, and 3 compared to arm 0 are displayed. Orange dotted lines represent the original data results.

SUPPLEMENTARY FIGURE 5

We performed 100 avatarizations of the WBCD datasets with the same parameters (*k*=20). For this dataset, we computed the F-score distribution of each variable. Results obtained with original data are then compared to those obtained with avatar data. F-scores are ranging around the original value and the order of importance is preserved. Such results imply that both strongly discriminating and poorly discriminating variables for prediction retained their properties.

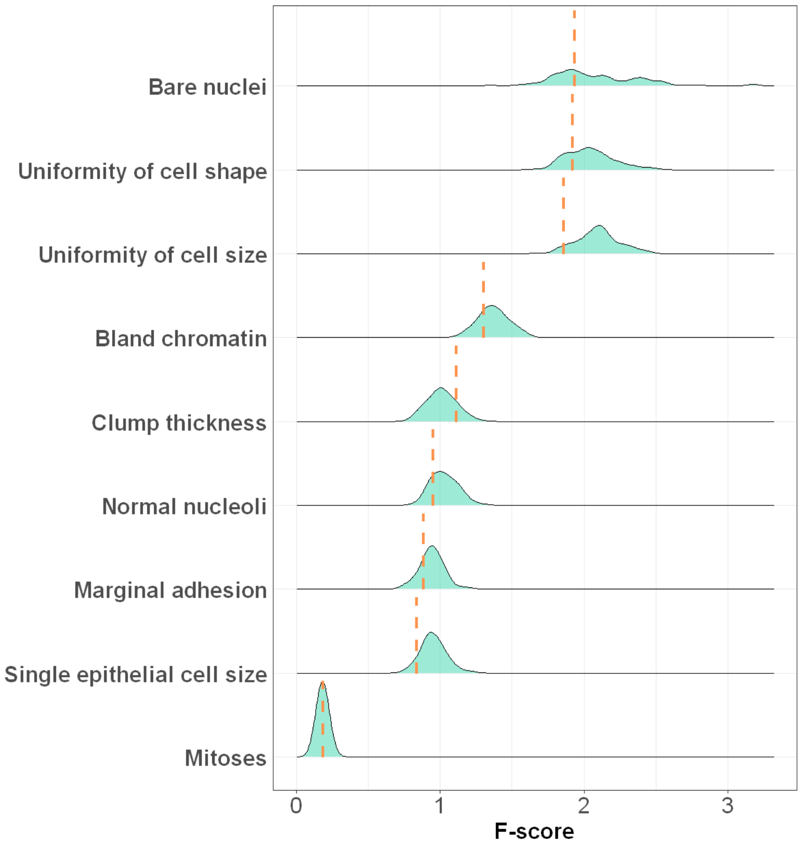

Figure 5: F-score distributions. 100 independent avatarizations of the WBCD dataset were performed (*k*=20). The distribution of the F-score computation for each variable compared with the variable "Class" is displayed. Orange dotted lines represent the original data results.

SUPPLEMENTARY FIGURE 6

Supplementary Figure 6 illustrates the DCR and NNDR metrics. The Distance to Closest Record (DCR) is the Euclidean distance between each synthetic record and its closest corresponding real neighbor. A high DCR implies a high level of privacy. The Nearest Neighbor Distance Ratio (NNDR), is the ratio between the distance of the closest and the distance of the second closest real neighbor for each synthetic record. It is bounded in [0, 1]. Higher values reveal better privacy. Low NNDR values indicate that each synthetic data can easily be associated with a unique original data, resulting in a low privacy level.

To provide a comparative framework for the DCR and NNDR metrics, the original data is divided in two sets. 70% of the dataset is used to create synthetic data. The holdout 30% is kept as original data to serve as a reference for the computation of DCR and NNDR.

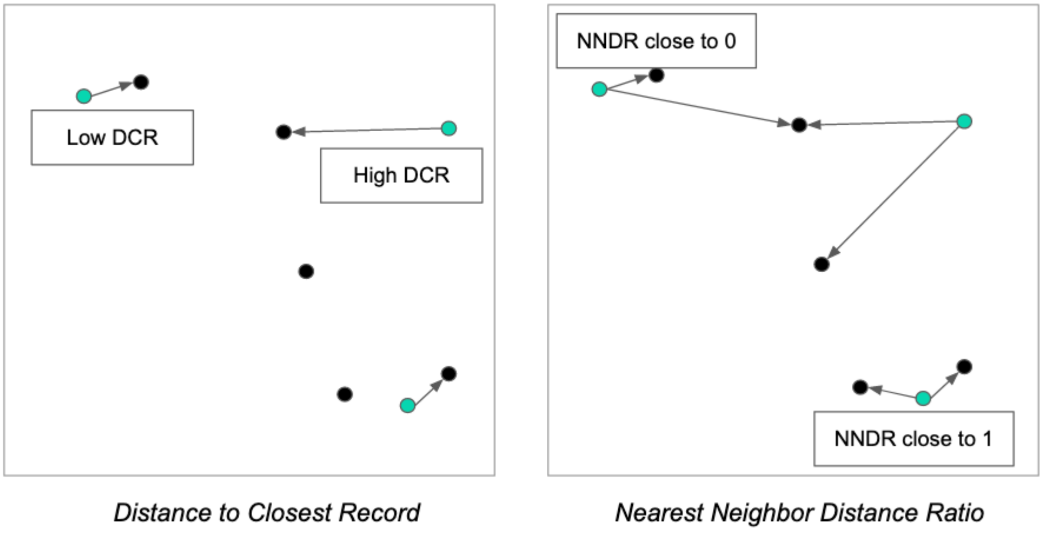

Figure 6: Visualization of DCR and NNDR

Legend: Original (black dots) and synthetic (green dots) records projected in a mathematical space for distance to closest (left) and nearest neighbor distance ratio (right) metric computation.

SUPPLEMENTARY FIGURE 7

Supplementary Figure 7 presents two cases of local cloaking. On the left, the local cloaking differs from zero, thus making it difficult to trace back the link between an individual and their avatar. On the right, the local cloaking is zero, thus making it easy to link an individual to their avatar. The denser the local environment, the more protected the individuals. The median of all local cloaking is indicative of the overall protection level. A higher local cloaking median suggests that the individual is more protected. We consider that a local cloaking beyond 5 can be reasonably estimated as a sufficient level of privacy. Similarly, a higher hidden rate indicates that an individual is more protected. For example, a hidden rate of 90% means that if an attacker associates the most similar avatar to one individual, they have a 90% chance of being wrong. We estimate that a value higher than 90% ensures a sufficient level of privacy. This metric is the percentage of sensitive individuals without a local cloaking at 0 (right side of the figure), i.e. the percentage of individuals whose closest avatar is not the avatar derived from their data.

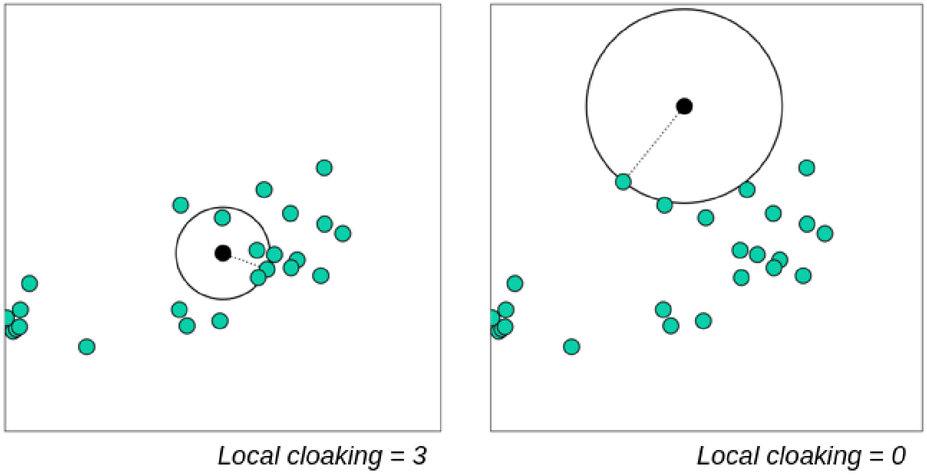

Figure 7: Visualization of local cloaking

Legend: One original individual (black dot) is surrounded by synthetized avatars (green dot). The link between the original individual and their generated avatar is indicated by a line. The plotted circle illustrates the count operated for the local cloaking metric computation. On the left side, the local cloaking equals three, on the right, it equals zero.

SUPPLEMENTARY METHOD 1

F-scores[1,2] enable measuring the discrimination of two sets with real numbers. Given training vectors of size m, x_k, for $k = 1, 2, \ldots$, m, if the number of positive and negative instances are n+ and n−, respectively, then the F-score of the ith feature is defined as:

$$F_i = \frac{(\bar{x}_i^{(+)} - \bar{x}_i)^2 + (\bar{x}_i^{(-)} - \bar{x}_i)^2}{\frac{1}{n_+ - 1}\sum_{k=1}^{n_+}(x_{k,i}^{(+)} - \bar{x}_i^{(+)})^2 + \frac{1}{n_- - 1}\sum_{k=1}^{n_-}(x_{k,i}^{(-)} - \bar{x}_i^{(-)})^2}, \quad (1)$$

where $\bar{x}_i$, $\bar{x}_i^{+}$, $\bar{x}_i^{-}$ are the average of the ith feature of the whole, positive, and negative datasets, respectively; $\bar{x}_{k,i}^{+}$ is the ith feature of the kth positive instance, k, i, and $\bar{x}_{k,i}^{-}$ is the ith feature of the kth negative instance. The numerator indicates the discrimination between the positive and negative sets; the denominator indicates the one within each of the two sets. Larger F-scores mean that the feature is more likely to be discriminative.

SUPPLEMENTARY METHOD 2

	Actual Positive	Actual Negative
Predicted Positive	True Positive (TP)	False Positive (FP)
Predicted Negative	False Negative (FN)	True Negative (TN)

$$\text{Accuracy (\%)} = \frac{TP+TN}{TP+FP+FN+TN} \times 100 \quad (2)$$

$$\text{Negative predictive value (\%)} = \frac{TN}{FN+TN} \times 100 \quad (3)$$

$$\text{Positive predictive value (\%)} = \frac{TP}{TP+FP} \times 100 \quad (4)$$

$$\text{Sensitivity (\%)} = \frac{TP}{TP+FN} \times 100 \quad (5)$$

$$\text{Specificity (\%)} = \frac{TN}{FP+TN} \times 100 \quad (6)$$

ROC curves are based on the values of true positives and false positives and therefore provide a trade-off between sensitivity and specificity. AUC is the area under the receiver operating characteristics curve.

SUPPLEMENTARY REFERENCE 1

1. Chen, Y.-W. & Lin, C.-J. Combining SVMs with Various Feature Selection Strategies. in *Feature Extraction* (eds. Guyon, I., Nikravesh, M., Gunn, S. & Zadeh, L. A.) vol. 207 315–324 (Springer Berlin Heidelberg, 2006).
2. Akay, M. F. Support vector machines combined with feature selection for breast cancer diagnosis. *Expert Syst. Appl.* **36**, 3240–3247 (2009).
